# Determinants of time to institutionalisation and related healthcare and societal costs in a community-based cohort of patients with Alzheimer’s disease dementia

**DOI:** 10.1007/s10198-018-1001-3

**Published:** 2018-09-03

**Authors:** Mark Belger, Josep Maria Haro, Catherine Reed, Michael Happich, Josep Maria Argimon, Giuseppe Bruno, Richard Dodel, Roy W. Jones, Bruno Vellas, Anders Wimo

**Affiliations:** 1 Lilly Research Centre, Eli Lilly and Company Limited, Erl Wood Manor, Sunninghill Road, Windlesham, Surrey GU20 6PH UK; 2 Parc Sanitari Sant Joan de Déu, CIBERSAM, Universitat de Barcelona, Sant Boi de Llobregat, Barcelona, Spain; 3 Agència de Qualitat i Avaluació Sanitàries de Catalunya (AQuAS), Barcelona, Spain; 4 Clinica della Memoria, Department of Neurology and Psychiatry, Sapienza University of Rome, Rome, Italy; 5 Geriatrie-Zentrum Haus Berge, Essen University Hospital, Essen, Germany; 6 RICE (The Research Institute for the Care of Older People), Royal United Hospital, Bath, UK; 7 Gérontopôle, Toulouse University Hospital, INSERM 1027, Toulouse, France; 8 Division of Neurogeriatrics, Department of Neurobiology, Care Sciences and Society, Karolinska Institutet, Stockholm, Sweden; 9 Centre for Research and Development, Uppsala University/County Council of Gävleborg, Gävle, Sweden

**Keywords:** Alzheimer’s disease, Societal costs, Institutionalisation, Predictors, Caregiver, I00 (Health Education and Welfare: General), I10 (Health: General), I19 (Health: Other)

## Abstract

**Objectives:**

To examine the costs of caring for community-dwelling patients with Alzheimer’s disease (AD) dementia in relation to the time to institutionalisation.

**Methods:**

GERAS was a prospective, non-interventional cohort study in community-dwelling patients with AD dementia and their caregivers in three European countries. Using identified factors associated with time to institutionalisation, models were developed to estimate the time to institutionalisation for all patients. Estimates of monthly total societal costs, patient healthcare costs and total patient costs (healthcare and social care together) prior to institutionalisation were developed as a function of the time to institutionalisation.

**Results:**

Of the 1495 patients assessed at baseline, 307 (20.5%) were institutionalised over 36 months. Disease severity at baseline [based on Mini-Mental State Examination (MMSE) scores] was associated with risk of being institutionalised during follow up (*p* < 0.001). Having a non-spousal informal caregiver was associated with a faster time to institutionalisation (944 fewer days versus having a spousal caregiver), as was each one-point worsening in baseline score of MMSE, instrumental activities of daily living and behavioural disturbance (67, 50 and 30 fewer days, respectively). Total societal costs, total patient costs and, to a lesser extent, patient healthcare-only costs were associated with time to institutionalisation. In the 5 years pre-institutionalisation, monthly total societal costs increased by more than £1000 (€1166 equivalent for 2010) from £1900 to £3160 and monthly total patient costs almost doubled from £770 to £1529.

**Conclusions:**

Total societal costs and total patient costs rise steeply as community-dwelling patients with AD dementia approach institutionalisation.

**Electronic supplementary material:**

The online version of this article (10.1007/s10198-018-1001-3) contains supplementary material, which is available to authorized users.

## Introduction

Cost-of-illness (COI) studies have shown that the two most important cost drivers in dementia care from a societal perspective are the costs of institutional care and the costs of informal care for patients who are cared for at home [1, 2].

Time to institutionalisation (also referred to as ‘nursing home placement’ or ‘transition into a care home’) is often used as an outcome measure in Alzheimer’s disease (AD) dementia trials. The median time to institutionalisation in dementia studies has been estimated to be between 30 and 40 months from study entry, but this depends on the severity of the patient’s disease at the time of study entry [3]. One of the goals of AD dementia management (including drug treatment) is to improve treatment in the community setting and stabilise the course of the disease, which may prolong the time to institutionalisation of people with dementia [4, 5]. This is especially true in high-income countries, where long-term institutional care constitutes a substantial part of care for people with dementia [6]. Delaying institutionalisation may increase the time patients with AD dementia can spend with their family and friends, improve quality of life and increase life expectancy [7]. It may also potentially be cost-saving from a societal perspective [5], although this is not fully supported by current evidence [8, 9].

Economic models of AD assessing long-term costs and outcomes generally use modelling techniques to extrapolate from short-term data because the follow-up periods in clinical trials are usually too short to measure parameters such as institutionalisation and death. To better estimate these models, detailed knowledge of the factors associated with patient care and societal costs is necessary. Time to institutionalisation could potentially be a significant determinant of societal costs in patients with AD dementia. However, it has only been used in a few model-based economic evaluations in AD dementia [10, 11]. There is a need for more data on the costs incurred during the pre-institutionalisation phase from a societal perspective to determine whether costs increase substantially for patients approaching institutionalisation. If this is the case, the view that institutionalisation is associated with large increases in costs could be challenged. To address this knowledge gap, it is important to have a complete set of input data for modelling the whole course of AD. There is also a need for better understanding of the factors that influence institutionalisation and of interventions that enable persons with AD dementia to remain in the community for longer.

Prospective observational studies such as the GERAS study, of the costs and resource use associated with community-dwelling patients with AD dementia and their caregivers [12], may provide useful long-term data on the time to institutionalisation in usual clinical practice as well as predictive factors and the relationship between time to institutionalisation and costs.

The aims of the current study were to estimate the healthcare and societal costs of caring for community-dwelling patients with AD dementia in relation to the time to institutionalisation. We present a proof-of-concept methodological approach for modelling disease progression and costs in patients with AD dementia for use in a cost-effectiveness model, using the GERAS population as an example. By applying the results of the GERAS study, we first assessed the cumulative incidence of institutionalisation over 36 months of follow up and identified factors associated with time to institutionalisation. Using these factors, we developed parametric models to estimate time to institutionalisation for all patients and then estimated healthcare and societal costs as a function of time to institutionalisation from the parametric models.

## Methods

### GERAS study design

GERAS was an 18-month prospective observational study of costs associated with the care of community-dwelling patients with AD dementia and their caregivers in three European countries (France, Germany, UK) [12]. The study design, patient characteristics and baseline costs have been reported in detail elsewhere [12].

The study enrolled community-dwelling patients aged at least 55 years, meeting the National Institute of Neurological and Communicative Disorders and Stroke/Alzheimer’s Disease and Related Disorders Association criteria for probable AD [13], with a Mini-Mental State Examination (MMSE) [14] score of ≤ 26, and presenting within the normal course of care. Patients were stratified by disease severity at baseline, as reported previously [12]: mild AD dementia (MMSE score = 21‒26), moderate AD dementia (MMSE score = 15‒20), moderately severe/severe (MS/S) AD dementia (MMSE score < 15). Ethical review board approval of the study was obtained in each country in accordance with individual country regulations. Written informed consent was obtained from all participants or their legal representative.

### Data collection

Data were collected at baseline and during routine care visits at 6, 12 and 18 months in all three countries and at 24, 30 and 36 months in France and Germany. Information collected for patients and caregivers included sociodemographics, comorbidities, medications, and health-related quality of life (HRQoL).

Patient cognitive function was assessed using the MMSE, with lower scores indicating reduced cognitive functioning. Functional ability was assessed using the Alzheimer’s Disease Cooperative Study Activities of Daily Living inventory (ADCS-ADL) [12, 15, 16], which was completed on behalf of the patient by his/her caregiver. The range for the total ADCS-ADL score is 0–78. Separate subscores were derived for basic activities of daily living (ADL; basic ADCS-ADL score range 0–22) and instrumental ADL (instrumental ADCS-ADL score range 0–56), with higher scores indicating better functioning for the total score and subscores [12]. Behavioural and psychological symptoms were assessed by the patient’s caregiver using the Neuropsychiatric Inventory (NPI)-12 [17] where a higher NPI-12 total score indicates more severe problems.

Information on healthcare resource use by patients and caregivers was collected using the Resource Utilization in Dementia (RUD) instrument [18] and several additional questions during interview. Resource use items collected included healthcare (medications, outpatient visits, hospital stays, emergency room visits, neuropsychological assessments), social care (community care services, such as district nurse, home aid, food delivery, day care, transportation, other; financial support, out-of-pocket expenses), changes to patient living accommodation (permanent, temporary; structural adaptations; institutionalisation), caregiver work status (working for pay [yes/no], lost work time) and caregiver time (i.e. time spent on giving informal care by the primary caregiver). Caregiver informal care time was recorded as time spent assisting the patient with basic ADL (e.g. bathing, feeding) and instrumental ADL (e.g. shopping, cooking), and was capped at 24 h/day. Details of other data collected but not used in the present analyses are available elsewhere [12].

### Cost estimation

Monthly costs values were estimated by applying country-specific unit costs of services and products (2010 values) to the healthcare and social care resource use data collected over the 36-month follow-up period. Full details of the unit costs applied and their sources are presented in Online Resource 1.

Total societal costs were evaluated using an opportunity cost approach taking into account productivity loss for working caregivers and lost leisure time for non-working caregivers. Costs were calculated for the month before each visit. Total societal costs were calculated by combining direct costs, consisting of patient healthcare costs (including medications, hospitalisations and outpatient visits) and patient social care costs (including community care services, structural adaptations, financial support and out-of-pocket expenses), and indirect costs, consisting of caregiver informal care costs (including the time spent giving care and missing work). Total patient costs were analysed as patient healthcare costs and patient social care costs combined. The list of resource use items collected in the section above reflects all cost categories included in the cost analyses.

The following imputation rules were applied for missing data: for institutionalised patients, mean monthly costs from the last visit were used for the period until institutionalisation and monthly costs for institutionalisation were used from institutionalisation up to 18 months for the UK and up to 36 months for France and Germany. For patients who died, last observation carried forward was used such that costs from the last known visit were extrapolated up to the date of death (no costs after death were computed). For patients with other reasons for discontinuation, the multiple imputation regression method [19] stratified by MMSE group and country was applied to missing costs. The list of factors used in the multiple imputation procedure was selected from those identified by Dodel et al. [20]. Fixed costs were applied to institutionalised patients (see Online Resource 1) and zero costs were applied once a patient died. Thus, multiple imputation was only used for patients with other reasons for discontinuation, including lost to follow up.

The unit cost of caregiver time for working caregivers was the value of lost production time based on the national average wage per country; for non-working caregivers, it was the value of lost leisure time based on 35% of the national average wage per country population (see Wimo et al. [12]). In the current analyses, French and German costs were converted from euros to pounds sterling using the conversion rate €1 = £0.8576 (calculated using the monthly exchange rate average for 2010 as reported previously [12]). The cost data are presented in pounds sterling because one aim of the study was to develop economic models for the National Institute for Health and Care Excellence (NICE) in the UK. However, overall monthly cost estimates are also given in euros (calculated using the conversion rate above).

### Time to institutionalisation

Time to institutionalisation was taken from the reasons for discontinuation from the study, where the date of institutionalisation was collected if the reason for discontinuation was reported as patient institutionalisation. The current analysis did not include temporary admission to an institution or full-time care at home based on caregiver time or responses to specific basic ADL questions. Time to institutionalisation was measured in days from the date of the baseline assessment to the date of institutionalisation.

### Statistical analyses

Demographics and baseline characteristics were summarised using descriptive statistics and were based on non-missing observations.

Competing risk analysis [21], where institutionalisation and death were considered as competing risks, was used to describe the cumulative incidence of institutionalisation during the 36-month follow-up period for the total study population.

Factors associated with time to institutionalisation were explored using Cox proportional hazards models of the 36-month data (18 months for the UK data); time to institutionalisation was censored at the time of last follow up or time to death for those subjects who did not report being institutionalised. The only competing risk in the Cox proportional hazards models was patient death, which was treated as a censored event (i.e. the patient was considered to have died before institutionalisation). One hundred different models using forward and backward selection were run, selecting 67% of subjects at random for inclusion in the model, and the factors identified in each model summarised. Entry and exclusion of individual factors was based on a significance level of 0.05. Patient characteristics considered in the models were age, gender, years of education, time since diagnosis of AD, comorbidities, and baseline scores for MMSE, total ADCS-ADL, instrumental ADCS-ADL, basic ADCS-ADL and NPI-12 total. Caregiver characteristics considered in the models were age, gender, relationship with patient (spouse yes/no), and caregiver working for pay (yes/no).

Additional analyses examined the effects of including AD medication as a factor in the model selection, or including the four subdomains of ADCS-ADL (basic activities, household activities, communication, outdoor activities) or four subdomains of NPI (psychosis, affective, apathy, hyperactivity) in the list of variables for consideration in the models, as well as interactions between the scores for cognition (MMSE) and function (ADCS-ADL). If the final selection suggested a subdomain rather than a main effect, then the models were compared based on their Akaike Information Criterion (AIC) and Bayesian Information Criterion (BIC) model fit statistics. If the difference was less than 3 then the model with total score rather than subdomain was used.

Any factor found to be significant in over 75% of the models was included in the parametric models used to predict time to institutionalisation. Again, the only competing risk in the parametric models was patient death, which was treated as a censored event in the analysis. To allow for different assumptions around the distribution of the data, the parametric models considered exponential, log-logistic, Weibull, log-normal and gamma distributions. Model fit was assessed using AIC and BIC model fit statistics, and the best fitting model was selected for use in the model that estimated societal and patient costs as a function of time to institutionalisation.

Models were fitted to estimate costs (*y*) as a function of time to institutionalisation (*x*). Separate models were developed for total societal costs, total patient costs (patient healthcare plus social care costs) and patient healthcare costs, determined using the cost category definitions described earlier. These three cost categories were chosen as they were considered to be the most important for payers. For each patient, the predicted time to institutionalisation (Pred_Inst) was calculated from the parametric model. Then, for each 6-month visit, the patient’s time to institutionalisation (Pre-Inst) was calculated as: Pre-Inst = Pred_Inst-visit. Each individual subject time point was treated as independent, had an associated cost and any missing cost visits used the imputation methods described earlier. The cost analysis only used data for the first 18 months so that data from all three countries could be used.

All data were analysed using SAS version 9.2 (SAS Institute, Cary, NC, USA).

## Results

The GERAS study included 1495 patients at baseline: 566 (37.9%) patients had mild AD dementia, 472 (31.6%) had moderate AD dementia and 457 (30.6%) had MS/S AD dementia. Table 1 presents the patient and caregiver characteristics at baseline for the overall cohort and by mild, moderate and MS/S AD dementia severity.

**Table 1:** Baseline characteristics of patients and caregivers in the overall study population and by AD dementia severity

Characteristics	Overall	Mild AD	Moderate AD	MS/S AD	*p* value^a^
Patient, *n*	1495	566	472	457	
Gender, *n* (%) female	819 (54.8)	271 (47.9)	269 (57.0)	279 (61.1)	< 0.001
Age, mean (SD)	77.6 (7.7)	77.3 (6.9)	77.8 (8.0)	77.6 (8.1)	0.934
Education, years, mean (SD)	10.4 (3.2)	11.1 (3.3)	10.1 (2.9)	10.0 (3.0)	< 0.001
Time since diagnosis of AD, years, mean (SD)	2.2 (2.2)	1.7 (2.0)	2.1 (2.0)	3.1 (2.4)	< 0.001
Patients with comorbidities, *n* (%)	1101 (73.6)	426 (75.3)	345 (73.1)	330 (72.2)	0.340
Number of comorbidities per patient, mean (SD)	1.4 (1.2)	1.5 (1.2)	1.4 (1.2)	1.4 (1.3)	0.608
MMSE total score^b^, mean (SD)	17.4 (6.3)	23.3 (1.6)	17.9 (1.7)	9.5 (4.3)	N/A
ADCS-ADL total score^c^, mean (SD)	46.5 (19.5)	58.4 (14.2)	48.3 (15.4)	29.9 (17.2)	< 0.001
Basic ADCS-ADL score^d^, mean (SD)	17.3 (5.2)	19.8 (3.1)	18.3 (3.8)	13.2 (6.0)	< 0.001
Instrumental ADCS-ADL score^e^, mean (SD)	29.1 (15.2)	38.5 (11.8)	29.9 (12.5)	16.6 (12.3)	< 0.001
NPI-12 total score^f^, mean (SD)	15.1 (15.3)	10.2 (10.7)	14.3 (12.6)	22.0 (19.4)	< 0.001
Caregiver, *n*	1493	565	472	456	
Gender, *n* (%) female	958 (64.2)	387 (68.5)	305 (64.6)	266 (58.3)	0.004
Age, mean (SD)	67.3 (12.0)	68.1 (11.6)	66.7 (11.7)	67.0 (12.9)	0.084
Relationship to patient, *n* (%)					0.024
Spouse	984 (65.9)	399 (70.6)	298 (63.1)	287 (62.9)	
Child	405 (27.1)	133 (23.5)	136 (28.8)	136 (29.8)	
Other	104 (7.0)	33 (5.8)	38 (8.1)	33 (7.2)	
Lives with patient, *n* (%)	1135 (76.0)	429 (75.9)	341 (72.2)	365 (80.0)	0.018
Working for pay, *n* (%)	355 (23.8)	133 (23.5)	120 (25.4)	102 (22.4)	0.457

All data are based on patients/caregivers with non-missing data (baseline characteristics data missing for two caregivers)

*AD* Alzheimer’s disease, *ADCS-ADL* Alzheimer’s Disease Cooperative Study Activities of Daily Living inventory, *MMSE* Mini-Mental State Examination, *MS*/*S* moderately severe/severe, *N*/*A* not applicable, *NPI* neuropsychiatric inventory, *SD* standard deviation

^a^*p* value for comparison between AD dementia severity groups (ANOVA for continuous variables, Cochran–Mantel–Haenszel for categorical variables)

^b^MMSE total score range 0‒26; enrolled patients were stratified according to baseline disease severity as having mild (MMSE 21–26 points), moderate (MMSE 15–20 points) or moderately severe/severe AD dementia (MMSE < 15 points)

^c^ADCS-ADL total score range 0‒78

^d^Basic ADCS-ADL score range 0‒22

^e^Instrumental ADCS-ADL score range 0‒56

^f^NPI-12 total score range 0‒144

Of the 1495 patients at baseline, 307 (20.5%) were institutionalised during the 36-month follow-up period: 122/419 (29.1%) patients from France, 91/550 (16.5%) patients from Germany, and 94/526 (17.9%) patients from the UK (18 months’ follow up for the UK). According to baseline AD dementia severity, the number (%) of patients institutionalised during follow up was 66 (11.7%), 111 (23.5%) and 130 (28.4%) in the mild AD, moderate AD and MS/S AD dementia groups, respectively. An additional 152 (10.2%) patients died overall before institutionalisation during the 36-month follow-up period. Other reasons for discontinuation included lost to follow up [57 (3.8%) patients], physician decision [32 (2.1%) patients], sponsor decision [1 (0.07%) patient], subject decision [206 (13.8%) patients] and subject entered clinical trial [2 (0.1%) patients].

**Fig. 1:**
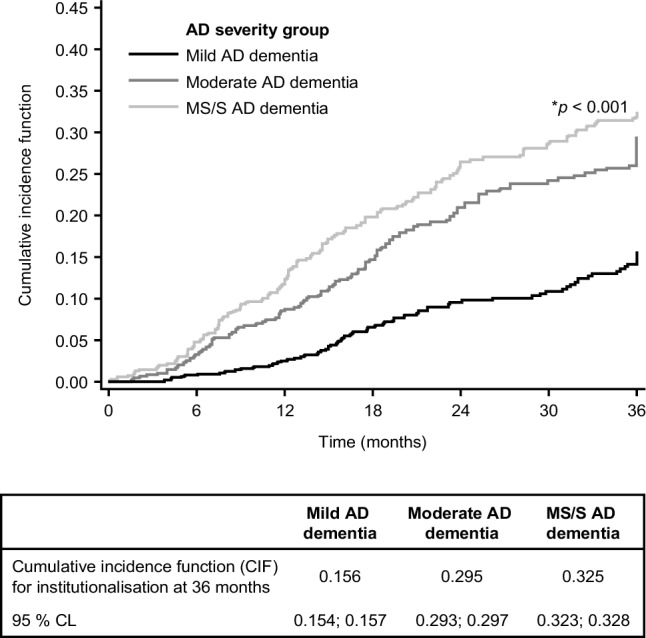
Cumulative incidence of institutionalisation over 36 months by AD dementia severity at baseline. *p < 0.001 for comparison across groups with mild, moderate and MS/S AD dementia (Wald test). *AD* Alzheimer’s disease, *CL* confidence limits, *MS*/*S* moderately severe/severe

Of the 1495 patients, only 11 were excluded from the time to institutionalisation analysis due to missing data. Figure 1 presents the cumulative incidence of institutionalisation over 36 months by AD dementia severity at baseline from the competing risk analysis. It shows that patients with greater disease severity at baseline have a higher risk of being institutionalised at 36 months; the Wald test for comparison across groups with mild, moderate and MS/S AD dementia was significant (*p* < 0.001).

Table 2 summarises the maximum likelihood parameter estimates of factors independently associated with time to institutionalisation from the 36-month data. Interpretation of regression coefficients shows that having a non-spousal caregiver was associated with a faster time to institutionalisation (944 fewer days to institutionalisation versus having a spousal caregiver), as was a worse baseline score in MMSE, instrumental ADCS-ADL and NPI-12 (67, 50 and 30 fewer days, respectively, for each one-point worsening in baseline score). However, a worse basic ADCS-ADL score at baseline was associated with a longer time to institutionalisation (89 days for each one-point change in score). Therefore, the following patient and caregiver factors were selected for inclusion in the final model to determine time to institutionalisation: MMSE total score at baseline, basic ADCS-ADL score at baseline, instrumental ADCS-ADL score at baseline, NPI-12 total score at baseline and caregiver relationship (spouse yes/no).

**Table 2:** Analysis of maximum likelihood parameter estimates of patient and caregiver factors associated with time to institutionalisation from the log-normal model of the 36-month data

Variables	Regression coefficient^a^	Standard error	95% confidence limits	*χ* ^2^	*p* value	Change in time to institutionalisation, days (months)^b^
Intercept	7.600	0.209	7.190; 8.010	1321.79	< 0.0001	–
MMSE total score^c^	0.034	0.009	0.016; 0.052	13.75	0.0002	− 67 (2.3)
Instrumental ADCS-ADL^c^	0.025	0.005	0.015; 0.036	23.44	< 0.0001	− 50 (1.7)
Basic ADCS-ADL^c^	− 0.044	0.014	− 0.071; − 0.017	10.13	0.0015	+ 89 (3.0)^d^
NPI-12 total score^c^	− 0.015	0.003	− 0.021; − 0.010	27.35	< 0.0001	− 30 (1.0)
Spousal caregiver, No (Ref = yes)	− 0.640	0.095	− 0.826; − 0.453	45.18	< 0.0001	− 944 (31.5)
Scale	1.206	0.054	1.105; 1.317	–	–	–

*ADCS-ADL* Alzheimer’s Disease Cooperative Study Activities of Daily Living inventory, *MMSE* Mini-Mental State Examination, *NPI* Neuropsychiatric Inventory

^a^The regression coefficient gives the change in time based on a one-point increase in the factor (for continuous factors) or the difference in time (for categorical factors) from the reference value. A one-point increase in score for MMSE and ADCS-ADL represents an improvement, while a one-point increase in NPI-12 score represents a worsening

^b^The method of calculating change in time to institutionalisation is available upon request. A negative sign indicates fewer days to institutionalisation. The interpretation is that a one-point worsening in MMSE score (i.e. a lower score) results in 67 fewer days (2.3 months) to institutionalisation; a one-point worsening in instrumental ADCS-ADL score (i.e. a lower score) results in 50 fewer days (1.7 months) to institutionalisation; a one-point worsening in basic ADCS-ADL score (i.e. a lower score) results in 89 more days (3.0 months) to institutionalisation; a one-point worsening in NPI-12 total score (i.e. a higher score) results in 30 fewer days (1.0 month) to institutionalisation; and patients with a non-spousal caregiver have 944 fewer days (31.5 months) to institutionalisation than those with spousal caregivers

^c^Score at baseline

^d^See “Discussion” for possible explanations for this finding

Additional analyses (data not shown) demonstrated that when AD medication was included as a factor, it did not enter into the final model. When the model selection included the baseline scores for the four ADCS-ADL subdomains (basic activities, household activities, communication, outdoor activities), the communication subdomain was selected and had lower AIC values than instrumental ADCS-ADL but the difference was not significant. When the NPI subdomains were included in the model, the NPI-12 total score was more predictive than if it were replaced with a specific subdomain.

**Fig. 2:**
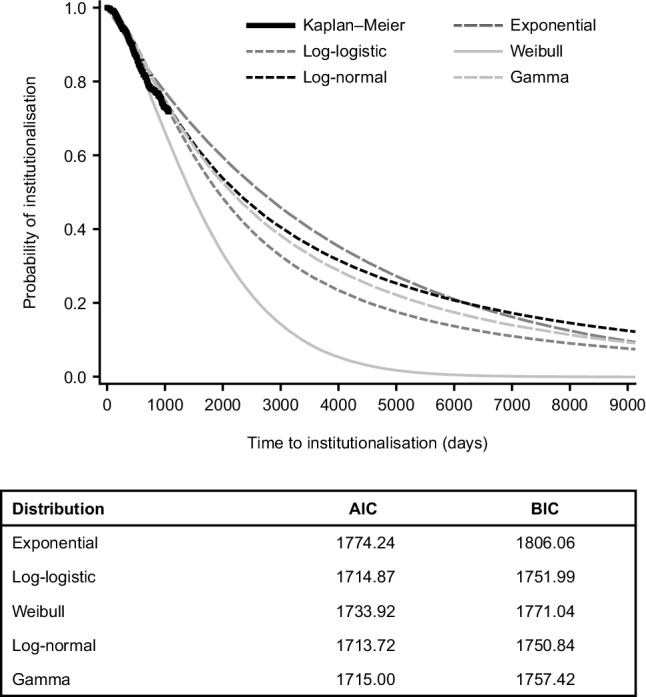
Extrapolation plots of time to institutionalisation beyond 36 months for the different model distributions examined in models of the 36-month data that included patient and caregiver factors. Data cut-off = 9125 days (25 years). *AIC* Akaike Information Criterion, *BIC* Bayesian Information Criterion

Figure 2 presents the time to institutionalisation extrapolated beyond 36 months to 9125 days (25 years; beyond the expected lifetime of the patients) for the different model distributions examined in models of the 36-month data that included patient and caregiver factors as described in Table 2. It shows that differences exist when extrapolating beyond the 36-month study period and highlights the impact associated with the choice of model. The models used suggest that, even after 10 years, not all patients would have been institutionalised. Model fits (based on AIC and BIC) were similar for the different distributions within the study period.

**Fig. 3:**
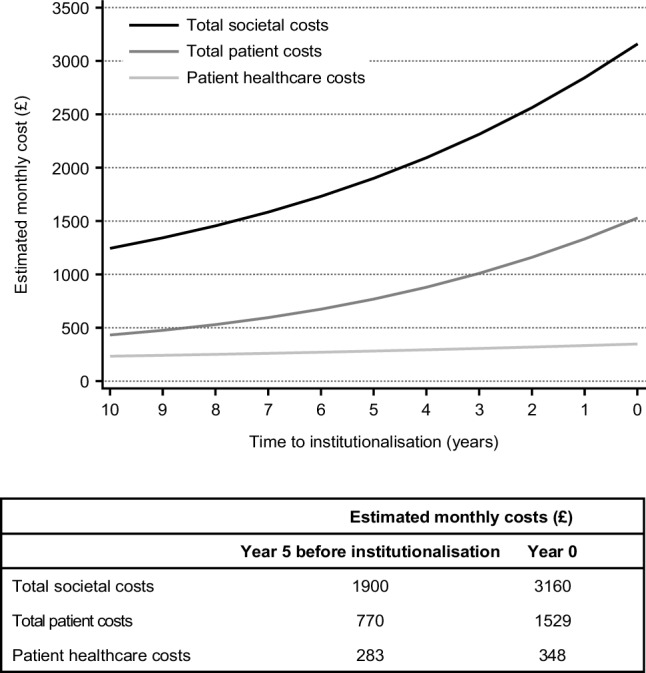
Estimated monthly total societal costs, total patient costs and patient healthcare costs by time to institutionalisation (years)

From the time-to-institutionalisation models, we chose the log-normal distribution to model the relationship between costs and time to institutionalisation as it had the best fit of the available data (lowest AIC and BIC values). Table 3 summarises the regression estimates from the log-normal models estimating costs (total societal costs, total patient costs, patient healthcare costs) as a function of time to institutionalisation. Country-specific regression estimates are presented in Online Resources 2–4. Overall, costs were imputed by multiple imputation for 298 (19.9%) patients: 118 (20.8%) in the mild AD dementia group, 85 (18.0%) in the moderate AD dementia group and 95 (20.8%) in the MS/S AD dementia group. Monthly total societal costs and total patient costs were associated with time to institutionalisation; these costs increased substantially in the 5 years prior to institutionalisation (Fig. 3). In the 5 years before institutionalisation, estimated monthly total societal costs increased by more than £1000 (€1166) from £1900 (€2216) to £3160 (€3685) and estimated monthly total patient costs almost doubled from £770 (€898) to £1529 (€1783) (see Fig. 3). Patient healthcare costs were associated with time to institutionalisation (Table 3) but increased relatively moderately from £283 (€330) to £348 (€406) in the 5 years before institutionalisation (Fig. 3).

**Table 3:** Estimates from the log-normal regression models of the association between costs and time to institutionalisation

	Estimate	Standard error	tValue	*p* value
Total societal costs^a^				
Intercept	3159.677	85.466	36.97	< 0.0001
Time to institutionalisation	− 334.025	38.541	− 8.67	< 0.0001
Time to institutionalisation^2^	18.572	4.851	3.83	0.0001
Time to institutionalisation^3^	− 0.434	0.174	− 2.49	0.0128
Total patient costs^a^				
Intercept	1528.964	54.984	27.81	< 0.0001
Time to institutionalisation	− 208.529	24.522	− 8.50	< 0.0001
Time to institutionalisation^2^	12.733	3.062	4.16	< 0.0001
Time to institutionalisation^3^	− 0.284	0.109	− 2.59	0.0095
Patient healthcare costs^a^				
Intercept	348.306	22.813	15.27	< 0.0001
Time to institutionalisation	− 14.883	6.223	− 2.39	0.0168
Time to institutionalisation^2^	0.347	0.346	1.00	0.3155

Time to institutionalisation (Pre-Inst in equations below) is in years

Estimates can be converted into the following equations:

EQ1: Total societal costs (£) = 3159.68 − (334.03 Pre-Inst) + (18.57 Pre-Inst^2^) − (0.43 Pre-Inst^3^)

EQ2: Total patient costs (£) = 1528.96 − (208.53 Pre-Inst) + (12.73 Pre-Inst^2^) − (0.28 Pre-Inst^3^)

EQ3: Patient healthcare costs (£) = 348.31 − (14.88 Pre-Inst) + (0.35 Pre-Inst^2^)

For example, using EQ1 and Pre-Inst = 3 to estimate monthly total societal costs at 3 years prior to institutionalisation:

Total societal costs (£) = 3159.68 − (334.03 × 3) + (18.57 × 3^2^) − (0.43 × 3^3^)

Total societal costs (£) = 3159.68 − (1002.09) + (167.13) − (11.61)

Total societal costs (£) = 2313.11

^a^The superscripts 2 and 3 refer to the quadratic and cubic terms, respectively, of the variable ‘time to institutionalisation’

## Discussion

Our results in community-dwelling patients with AD dementia from the GERAS study showed that, during the 5 years prior to institutionalisation, estimated monthly total societal costs increased steeply and total patient costs almost doubled, without a substantial rise in patient healthcare costs (i.e. direct medical costs including hospitalisations, visits to general practitioners and specialists, and medication). These findings reflect escalating caregiver informal care costs and patient social care costs as a patient approaches institutionalisation, which are probably due to increasing patient dependence resulting from worsening disease severity.

The difference between total societal costs and total patient costs is likely a result of caregiver informal care costs, which are the main component of societal costs [12] and remain so irrespective of differences in the unit costs used to calculate the different components of informal care costs, such as country-specific wages [12, 20, 22]. Informal care is largely driven by the functional ability of the patient, and greater impairment in ADL is an important predictor of societal costs [23, 24]. Our finding that total societal costs rise steeply as community-living patients approach institutionalisation suggests that institutionalisation per se may not result in a huge increase in societal costs when caregiver informal care costs are included. However, institutionalisation results in a shift from unpaid informal care to a direct cost for the provider of institutional care, and there are few data available on resource use and costs by category following institutionalisation [2]. Our findings are consistent with a recent cross-sectional study in Germany, which showed that the societal costs of care for dementia patients living in the community were higher than those for institutionalised patients after controlling for various factors including functional impairment and when including a value for informal care [25]. However, a study in eight European countries of the costs of caring for people with dementia showed that monthly societal costs for recently institutionalised patients were almost twofold higher than those for patients receiving home care [5]. Although the authors speculated that delaying institutionalisation may be cost-saving from a societal perspective, they pointed out that this depends on the country, the value given to informal caregiver time and the level of patient impairment [5]. Patients with a very low level of ADL independence in the community-care setting might have higher costs compared to those in the institutional care setting; thus, delaying institutionalisation for such patients would not be cost-reducing. Using baseline data from the GERAS study, Reed et al. [26] showed that higher total societal costs were associated with impairment in all aspects of ADL and that patient healthcare and social care costs were associated with total ADL and basic ADL but not instrumental ADL.

A recent insurance claims data analysis of patients with dementia in Germany showed that healthcare service utilisation (e.g. physician visits, inpatient days, drug prescriptions) and related costs increased in the 3 months leading up to institutionalisation and then stabilised or decreased [27]. That study took a payer’s perspective and did not include a value for informal care in the community setting. Moreover, the authors stated that institutional care might be the less costly option from a societal perspective. In our study, patient healthcare costs represented only a small proportion of the total societal costs [12] and increased only moderately during the 5 years before institutionalisation. The difference between our study and the German insurance claims study in the pattern of healthcare costs prior to institutionalisation could have been due to specific characteristics of the GERAS population, a greater likelihood that GERAS study participants were already relatively high-cost patients when they entered the study (patients were required to have an informal caregiver to enter GERAS, which would incur associated costs [12, 20, 22]), differences between countries in care provision, or that the increasing care needs of the patients were being met by caregivers or social care. Patient healthcare costs are not dependent on patient AD severity; in each of the countries participating in the GERAS study, patient healthcare costs did not differ across AD severity groups at baseline [12].

### Factors associated with institutionalisation

The regression model found that having a non-spousal caregiver, poorer cognition (MMSE), greater functional impairment in instrumental ADL and worse behavioural disturbance (NPI-12) were all independently associated with a faster time to institutionalisation over 36 months.

Yaffe et al. [4] showed that multivariate models which combine patient and caregiver factors are better able to predict time to institutionalisation than a model including patient factors alone. In our analysis, caregiver relationship had the greatest impact on time to institutionalisation. Our results showing that having a non-spousal caregiver was associated with a faster time to institutionalisation (over 2.5 years compared with having a spousal caregiver) are in line with those of Hébert et al. [28] who reported that having a spouse as a caregiver was a protective factor. Additionally, in their systematic review, Luppa et al. [3] showed earlier institutionalisation among patients when their caregiver is a child or other relative rather than a spouse. Moreover, in their study of community-dwelling patients with AD, Soto et al. [29] found that living alone or with family presented a higher risk of institutionalisation than living with a spouse. Further studies have found that patients who live alone have an increased risk of institutionalisation compared with those who live with others [3, 30, 31]. Additional caregiver factors that may affect time to institutionalisation of patients with AD dementia include caregiver age, health, depression and burden [3, 4, 28, 31]. Studies have shown earlier institutionalisation among patients when their caregivers are employed, have a higher level of education or have a higher income [3]. Caregiver factors associated with institutionalisation of people with AD in the LASER-AD study were having a paid versus family carer, and the carer being less educated and spending fewer hours caring [32].

Our finding that several patient factors are associated with time to institutionalisation is in accordance with previous research. There is now considerable evidence that poorer cognition and lower functional abilities at baseline are associated with earlier institutionalisation of patients with dementia [3, 32‒35]. Most studies assessed overall functional ability (i.e. basic and instrumental ADL), although some assessed instrumental ADL separately [33, 35]. A deterioration in instrumental ADL is usually observed in patients with AD before a decline in basic ADL [36]. In a modelling analysis of data from a randomised controlled trial of AD patients receiving rivastigmine or donepezil treatment for up to 2 years, Hatoum et al. [37] showed that lower baseline ADL scores (total, basic and instrumental ADL) were all independently associated with a faster time to institutionalisation. In our analysis, both basic and instrumental ADL scores were included in the multivariate model. It is unclear why a worse baseline basic ADCS-ADL score was associated with a longer time to institutionalisation in our study, whereas a worse baseline instrumental ADCS-ADL score was associated with a faster time to institutionalisation, but there may be several reasons. First, because there is a high correlation between cognition and function, when both factors are included in the same model, one of the variables moves in the opposite direction compared with when it is fitted independently [24]. Thus, the inclusion of baseline MMSE score in our model may have influenced the association between baseline basic ADCS-ADL score and time to institutionalisation. Another possible explanation for our finding that a worse baseline basic ADCS-ADL score was associated with a longer time to institutionalisation may be that the patients were already incapacitated and highly dependent on their caregiver, but were well supported and receiving more community-based services.

Our result showing that worse patient behavioural problems at baseline were associated with a faster time to institutionalisation is also consistent with some previous research [30, 34, 38, 39]. Although some analyses found that different NPI domains or individual neuropsychiatric symptoms were more relevant [34, 39], the NPI-12 total score was the most important in our analysis. Other studies have found that patient sociodemographics are associated with institutionalisation: patients who are older or male have an increased risk of and/or shorter time to institutionalisation, whereas those with a higher level of education have a longer time to institutionalisation [3].

In a 3-year prospective naturalistic study in Sweden, multivariate analyses showed that worse baseline MMSE and instrumental ADL scores were associated with a faster time to institutionalisation, as were the patient living alone, a lower versus higher dose of acetylcholinesterase inhibitor, an increase of more than 7 h per week of home help services and an increase of more than 3 days per week of adult day care [35]. When AD treatment was included in our additional analysis of further potential factors in the model selection, it did not enter the final model, meaning that use of acetylcholinesterase inhibitors or memantine was not shown to be associated with time to institutionalisation. Randomised controlled trials have provided conflicting results regarding delaying institutionalisation with acetylcholinesterase inhibitors and/or memantine in community-living patients with AD [40, 41]. Also, in an observational study in the USA, Lopez et al. [42] found that treatment with an acetylcholinesterase inhibitor alone or in combination with memantine prolonged the time to institutionalisation of patients with AD compared with untreated patients. The data from the Lopez study have been applied to economic models in several countries and have shown that combination therapy is cost-saving from a societal perspective [43, 44].

Importantly, we observed differences in the time to institutionalisation when extrapolating beyond the 36-month study period using models with different distributions (see Fig. 2). This demonstrates uncertainty around long-term outcomes and indicates the need for more long-term follow-up data [45] and sensitivity analyses, as recommended by NICE [46]. Our results highlight the importance of choosing the correct extrapolation model, since the use of different models can produce very different results [46].

This methodology could be applied to country-specific cost-effectiveness models through the use of country-specific data sources (as shown in Online Resources 2–4).

### Strengths and limitations

The study has a number of strengths. It consisted of a large, community-based sample of patients with AD, and included patients across a wide range of AD severity, assessed using standardised instruments and followed for up to 3 years. We also used a transparent methodology for our cost estimations. Only 11 patients were excluded from the time to institutionalisation analysis due to missing data. The numbers of missing patients in the cost analyses were not related to the severity of disease, as the proportion of missing patients was similar in each disease severity group.

However, there are some limitations. First, in the models used, we assumed a log-normal distribution when extracting beyond the 36-month observational period. However, as can be seen in Fig. 2, the choice of distribution will affect the slope of these curves. Other distributions should be included in sensitivity analyses when using this methodology for cost-effectiveness modelling. In addition, sensitivity analyses involving other types of costing methods, such as replacement costs, should be performed. Longer term follow-up data on time to institutionalisation, which are continuing to be collected, will reduce the uncertainty introduced when extrapolating beyond the study period. Also, the parametric survival model may not fit the data perfectly. Second, patient age was not included in our final model because it was a non-significant covariate, but it has been associated with a faster time to institutionalisation in other studies [3]. Patient age may have been excluded from the final model due to inclusion of the caregiver relationship, i.e. older patients are more likely to have a non-spousal caregiver [47]. Also, we did not control for the progression of dementia, which could be achieved by calculating a measure that displays differences in cognitive functioning or status of independence. It is feasible that costs could be driven by fast progressing dementia while other non-problematic trajectories remain undiscovered. Third, we analysed combined data from three high-income European countries, but time to institutionalisation for patients in each country will be influenced by the availability of long-term care institutions and resources, and national policies on the organisation of dementia care [8, 48‒50]. In low- and middle-income countries, where resources are scarce, institutionalisation rates are consequently low [50]. There are also cultural differences between and within countries in the preferences for institutional care, which limits the generalisability of our findings. Moreover, institutionalisation may occur for many reasons, which may vary between countries [51], and for reasons that may be unrelated to AD. Fourth, the risk of, or time to, institutionalisation may be affected by non-pharmacological interventions or other variables not collected as part of the GERAS study [52], but information on any such interventions in patients and/or caregivers was not collected. The risk of institutionalisation will also change over time, as shown by Howard et al. [41]. The variables associated with institutionalisation will also vary with time, but we only included baseline variables in our analyses. Long-term data from the GERAS study could be used to explore the risks of institutionalisation and effects on costs. Fifth, there is possible selection bias due to the recruitment of the study participants mostly from memory clinics, which may limit the generalisability of the findings as the sample is not fully representative of all AD patients living in the community. Furthermore, the role of family caregivers does not finish upon institutionalisation of their relative; they may continue to experience stress and financial pressures after the transition to institutional care [53]; this should be considered when taking a societal approach to costs. As we did not estimate costs after institutionalisation, we could not compare costs before and after institutionalisation.

## Conclusions

This proof-of-concept model demonstrates that total societal costs and total patient costs rise steeply as community-dwelling patients with AD dementia approach institutionalisation, whereas patient healthcare costs increase only moderately. The main cost impact is from caregivers and community services, which varies according to patient functional ability and dependence. Caregiver factors are important in understanding the risk of institutionalisation in community-living patients with AD dementia. The key caregiver factor in this community-based cohort is being a non-spouse of the patient, which was associated with a faster time to institutionalisation (over 2.5 years). The implications of this finding are that we may need to invest in better community support for caregivers who want to keep patients with AD at home, although this will be more costly from a societal perspective. There is a need for long-term data to fully understand the patterns and costs of institutionalisation.

## Electronic supplementary material

Below is the link to the electronic supplementary material.

Supplementary material 1 (DOCX 29 KB)

Supplementary material 2 (DOCX 29 KB)

Supplementary material 3 (DOCX 49 KB)

Supplementary material 4 (DOCX 29 KB)
