# Supplementary material for: Determinants of time to institutionalisation and related healthcare and societal costs in a community-based cohort of patients with Alzheimer’s disease dementia
Source: Eur J Health Econ. 2018 Sep 3;20(3):343–55. doi: 10.1007/s10198-018-1001-3 (PMC6438944; doi:10.1007/s10198-018-1001-3)
Supplement: Supplementary file 1 — Supplementary material 1 (DOCX 29 KB) [file 10198_2018_1001_MOESM1_ESM.docx]

**Title:** Determinants of time to institutionalisation and related healthcare and societal costs in a

community-based cohort of patients with Alzheimer’s disease dementia

**Authors:** Mark Belger, Josep Maria Haro, Catherine Reed, Michael Happich, Josep Maria Argimon, Giuseppe Bruno, Richard Dodel, Roy W. Jones, Bruno Vellas, Anders Wimo

**Corresponding author:** Mark Belger, Erl Wood Manor, Sunninghill Road, Windlesham, Surrey, GU20 6PH, email: [belger_mark@lilly.com](mailto:belger_mark@lilly.com)

**Online Resource 2** Cost estimates (in €) from log-normal regression models of the association between costs and time to institutionalisation in France

|  | **Estimate** | **Standard error** | ***p* value** |
| --- | --- | --- | --- |
| ***Total societal costs^a^*** |  |  |  |
| Intercept | 3855.15 | 150.04 | <0.0001 |
| Time to institutionalisation | -619.20 | 76.54 | <0.0001 |
| Time to institutionalisation^2^ | 47.82 | 10.89 | <0.0001 |
| Time to institutionalisation^3^ | -1.34 | 0.44 | 0.002 |
| ***Total patient costs^a^*** |  |  |  |
| Intercept | 1719.27 | 70.83 | <0.0001 |
| Time to institutionalisation | -191.80 | 21.89 | <0.0001 |
| Time to institutionalisation^2^ | 6.41 | 1.38 | <0.0001 |
| ***Patient healthcare costs^a^*** |  |  |  |
| Intercept | 607.03 | 51.27 | <0.0001 |
| Time to institutionalisation | -54.76 | 15.85 | 0.0006 |
| Time to institutionalisation^2^ | 1.85 | 1.00 | 0.06 |

Time to institutionalisation (Pre-Inst in equations below) is in years

^a^The superscripts 2 and 3 refer to the quadratic and cubic terms, respectively, of the variable ‘time to institutionalisation’

Estimates can be converted into the following equations:

EQ1: Total societal costs (€) = 3855.15 – (619.20 Pre-Inst) + (47.82 Pre-Inst^2^) – (1.34 Pre-Inst^3^)

EQ2: Total patient costs (€) = 1719.27 – (191.80 Pre-Inst) + (6.41 Pre-Inst^2^)

EQ3: Patient healthcare costs (€) = 607.03 – (54.76 Pre-Inst) + (1.85 Pre-Inst^2^)
