# Supplementary material for: Determinants of time to institutionalisation and related healthcare and societal costs in a community-based cohort of patients with Alzheimer’s disease dementia
Source: Eur J Health Econ. 2018 Sep 3;20(3):343–55. doi: 10.1007/s10198-018-1001-3 (PMC6438944; doi:10.1007/s10198-018-1001-3)
Supplement: Supplementary file 2 — Supplementary material 2 (DOCX 29 KB) [file 10198_2018_1001_MOESM2_ESM.docx]

**Title:** Determinants of time to institutionalisation and related healthcare and societal costs in a community-based cohort of patients with Alzheimer’s disease dementia

**Authors:** Mark Belger, Josep Maria Haro, Catherine Reed, Michael Happich, Josep Maria Argimon, Giuseppe Bruno, Richard Dodel, Roy W. Jones, Bruno Vellas, Anders Wimo

**Corresponding author:** Mark Belger, Erl Wood Manor, Sunninghill Road, Windlesham, Surrey, GU20 6PH, email: [belger_mark@lilly.com](mailto:belger_mark@lilly.com)

**Online Resource 3** Cost estimates (in €) from log-normal regression models of the association between costs and time to institutionalisation in Germany

|  | **Estimate** | **Standard error** | ***p* value** |
| --- | --- | --- | --- |
| ***Total societal costs^a^*** |  |  |  |
| Intercept | 3743.01 | 147.34 | <0.0001 |
| Time to institutionalisation | -193.47 | 28.84 | <0.0001 |
| Time to institutionalisation^2^ | 3.21 | 1.18 | 0.007 |
| ***Total patient costs^a^*** |  |  |  |
| Intercept | 2036.63 | 124.73 | <0.0001 |
| Time to institutionalisation | -286.40 | 40.27 | <0.0001 |
| Time to institutionalisation^2^ | 18.34 | 3.67 | <0.0001 |
| Time to institutionalisation^3^ | -0.40 | 0.09 | <0.0001 |
| ***Patient healthcare costs^a^*** |  |  |  |
| Intercept | 472.57 | 62.79 | <0.0001 |
| Time to institutionalisation | -11.69 | 12.29 | 0.34 |
| Time to institutionalisation^2^ | 0.20 | 0.50 | 0.69 |

Time to institutionalisation (Pre-Inst in equations below) is in years

^a^The superscripts 2 and 3 refer to the quadratic and cubic terms, respectively, of the variable ‘time to institutionalisation’

Estimates can be converted into the following equations:

EQ1: Total societal costs (€) = 3743.01 – (193.47 Pre-Inst) + (3.21 Pre-Inst^2^)

EQ2: Total patient costs (€) = 2036.63 – (286.40 Pre-Inst) + (18.34 Pre-Inst^2^) – (0.40 Pre-Inst^3^)

EQ3: Patient healthcare costs €) = 472.57 – (11.69 Pre-Inst) + (0.20 Pre-Inst^2^)
