# Supplementary material for: Determinants of time to institutionalisation and related healthcare and societal costs in a community-based cohort of patients with Alzheimer’s disease dementia
Source: Eur J Health Econ. 2018 Sep 3;20(3):343–55. doi: 10.1007/s10198-018-1001-3 (PMC6438944; doi:10.1007/s10198-018-1001-3)
Supplement: Supplementary file 3 — Supplementary material 3 (DOCX 49 KB) [file 10198_2018_1001_MOESM3_ESM.docx]

**Title:** Determinants of time to institutionalisation and related healthcare and societal costs in a community-based cohort of patients with Alzheimer’s disease dementia

**Authors:** Mark Belger, Josep Maria Haro, Catherine Reed, Michael Happich, Josep Maria Argimon, Giuseppe Bruno, Richard Dodel, Roy W. Jones, Bruno Vellas, Anders Wimo

**Corresponding author:** Mark Belger, Erl Wood Manor, Sunninghill Road, Windlesham, Surrey, GU20 6PH, email: [belger_mark@lilly.com](mailto:belger_mark@lilly.com)

**Online Resource 1.** Unit costs and source of resource utilisation items for (a) France, (b) Germany, and (c) UK

**(a) France**

| **Unit cost** | **Unit of measurement** | **Unit cost (€; 2010 values)** | **Source** |
| --- | --- | --- | --- |
| **Caregiver informal care costs** | | | |
| Caregiver time: lost production | Hour | 24.27 | Average gross wage for country  INSEE: <http://www.insee.fr/fr/ffc/ipweb/ip1300/ip1300.pdf>, Accessed on September 26, 2011.  OECD Employer social security contributions: [www.oecd.org/ctp/taxdatabase](http://www.oecd.org/ctp/taxdatabase), Accessed on September 26, 2011. |
| Caregiver time: lost leisure time | Hour | 8.50 | 35% of average gross wage |
| Cost of caregiver missing work | Hour | 24.27 | As average gross wage |
| **Healthcare resource use costs** | | | |
| *Nights in hospital* |  |  |  |
| Geriatric ward | Night | 278.19 | ATIH: <http://www.atih.sante.fr/openfile.php?id=2411>, Accessed on September 26, 2011. |
| Psychiatric ward | Night | 310.2 | Source as above |
| Internal medicine ward | Night | 279.92 | Source as above |
| Surgical ward | Night | 673.73 | Source as above |
| Other | Night | 385.51 | Assumed as average of above values. |
| *Emergency room (ER) visits* | Visit | 144.11 | Ministry of Labour, Employment and Health of France (70% reimbursement): <http://www.sante.gouv.fr/IMG/pdf/Rappel_des_enjeux_des_modalites_des_schemas_cibles_et_transitoires.pdf>, Accessed on September 26, 2011. |
| *Outpatient visits* |  |  |  |
| General practitioner | Visit | 23.89 | Généraliste secteur 1. Tariff €26, rate reimbursement 70%, amount paid €17.2:  l'Assurance Maladie: <http://www.ameli.fr/assures/soins-et-remboursements/combien-serez-vous-rembourse/consultations/les-consultations-en-metropole/dans-le-cadre-du-parcours-de-soins-coordonnes_ain.php>, Accessed on April 20, 2011. |
| Geriatrician | Visit | 44.04 | Psychiatre Neuropsychiatre Neurologue secteur 1. Average of three types of tariffs: tariff €57, €43, €37, rate reimbursement 70%  Source as above |
| Neurologist | Visit | 44.04 | Psychiatre Neuropsychiatre Neurologue secteur 1. Average of three types of tariffs: tariff €57, €43, €37, rate reimbursement 70%  Source as above |
| Psychiatrist | Visit | 44.04 | As above for geriatrician/neurologist |
| Physiotherapist | Visit | 17.15 | Tariff = AMK (€2.04) page 22 * coefficient of care. Assumed as re-education of gait in the elderly (Article 9 -Rééducation de la déambulation du sujet âgé”, page 77), coefficient = 8. Au total, cost per visit = 2.04*8 = €16.32 (global cost), rate reimbursement 60%: NGAP: <http://orthoptie.net/textes/NGAP.pdf>, Accessed on September 26, 2011. |
| Occupational therapist | Visit | 17.15 | As above for physiotherapist |
| Social worker | Visit | 32.58 | Based on cost for UK. Assumed 30 minute visit.  Unit Costs of Health and Social Care 2010: [www.pssru.ac.uk/pdf/uc/uc2010/uc2010_s01.pdf](http://www.pssru.ac.uk/pdf/uc/uc2010/uc2010_s01.pdf), Accessed on September 26, 2011. |
| Psychologist | Visit | 50.92 | Dossier familial: <http://www.dossierfamilial.com/sante-psycho/psycho/combien-coute-une-psychotherapie,503>, Accessed on September 26, 2011.  Cost varies depending on clinic and is not reimbursed. This cost is a mean estimate. |
| Other healthcare professional | Visit | 34.46 | Assumed as average of above costs, excluding social worker visits. |
| **Community care services** |  |  |  |
| District nurse | Visit/hour | 21.01 | Page 57 / Mean cost per visit  For all interventions in dementia, the total average cost per visit of SSAID interventions at a patient’s home is equal to €20 (60% reimbursement):  Les patients en service de soins infirmiers à domicile (SSIAD): <http://www.travail-emploi-sante.gouv.fr/IMG/pdf/SSIAD.pdf>, Accessed on September 26, 2011. |
| Home support worker | Visit/hour | 8.95 | The salary of a home support worker varies in Smic from €1,392 to €1,450 per month (Statut de la fonction publique territorial). For those who work part time, wages are based on the Smic hourly rate (€9.18/hour).  CIDJ: <http://www.cidj.com/metier.aspx?docid=286&catid=1>, Accessed on June 10, 2011. |
| Home-delivered meals | Visit/hour | 8.74 | Individual tariff €8.59 Domicile Service Plus €8.5 plus €1 supplement.  Portage de repas a domicile: <http://www.ville-guerande.fr/vivre/services-la-personne/personnes-agees-vivre-domicile#Portage%20de%20repas%20à%20domicile>, Accessed on September 26, 2011. |
| Transportation | Visit/hour | 5.29 | EHPAD: <http://www.clic-rouen.fr/article.php3?id_article=39>, Accessed on September 26, 2011. Assumed average distance to community care center is 10 km (total travel distance =10*2 = 20 km).  Private car: €0.26/km; Ambulance: €52 (trip <5 km) or €2/cm (trip >5 km); Nonemergency medical transport:  €0.78/km |
| Day care | Visit/hour | 13.04 | Based on cost for UK |
| Other services | Visit/hour | 11.00 | Estimated as average cost of other community care services |
| **Cost of medications for other disorders** |  |  |  |
| Depression | Day | 0.37 | Average cost of three most common treatments (based on a Swedish database of drug prescriptions)  Medication lists: IMS MIDAS quarterly sales data based on French DOT market share for 2010  Medication costs: BdM_IT > Recherche par code. (<http://www.codage.ext.cnamts.fr/codif/bdm_it/index.php?p_site=AMELI>, Accessed on April 19, 2011)  DDD: ATC/DDD Index 2011 (<http://www.whocc.no/atc_ddd_index/>, Accessed on April 19, 2011)  DDD adjusted for elderly dosing BNF 62 |
| Epilepsy | Day | 1.84 | As above (except the adjustment for elderly dosing) |
| Stroke | Day | 0.38 | As above (except the adjustment for elderly dosing) |
| Ischemic cardiac | Day | 0.25 | As above (except the adjustment for elderly dosing) |
| Hypertension | Day | 0.29 | As above (except the adjustment for elderly dosing) |
| Diabetes mellitus | Day | 0.28 | As above (except the adjustment for elderly dosing) |
| Hypercholesterolemia | Day | 0.78 | As above (except the adjustment for elderly dosing) |
| Urinary tract disorder | Day | 0.45 | Based on cost for tamsulosin [tamsulosine biogaran LP 0.4 mg, extended-release capsules (pack of 30)]  Sources as above |
| Obstructive pulmonary | Day | 0.37 | Based on cost for Pulmicort Turbuhaler [Pulmicort Turbuhaler 400 µg/dose (budesonide)] Recipient dosing with mouthpiece (100 doses), powder for oral inhalation;  Average cost based on Table 1 in Piperno D, Huchon G, Pribil C, Boucot I, Similowski T (2003) The burden of COPD in France: results from the Confronting COPD survey. *Respir Med* **97**(Suppl C),S33-42; values inflated to 2010 values  Other sources as above |
| **AD medications** |  |  |  |
| Donepezil | Day | 4.12 | Based on DDD from WHO: ATC/DDD Index 2011 (<http://www.whocc.no/atc_ddd_index/>, Accessed on April 19, 2011)  Medication costs from a French database: BdM_IT > Recherche par code. (<http://www.codage.ext.cnamts.fr/codif/bdm_it/index.php?p_site=AMELI>, Accessed on April 19, 2011) |
| Galantamine | Day | 2.77 | Sources as above |
| Memantine | Day | 3.23 | Sources as above |
| Rivastigmine | Day | 3.05 | Sources as above |
| Other AD medication | Day | 3.29 | Assumed as average of above costs. |
| **Psychiatric/hypnotic medication** |  |  |  |
| Antidepressants | Day | 0.38 | Based on average cost for three most common drugs used.  Medication lists: IMS MIDAS quarterly sales for France in 2010 market share per DOT  Medication costs: BdM_IT > Recherche par code. (<http://www.codage.ext.cnamts.fr/codif/bdm_it/index.php?p_site=AMELI>, Accessed on April 19, 2011)  DDD: ATC/DDD Index 2011 (<http://www.whocc.no/atc_ddd_index/>, Accessed on April 19, 2011)  DDD adjusted for elderly dosing: BNF 62 |
| Antipsychotics | Day | 0.08 | As above |
| Anxiolytics | Day | 0.01 | As above |
| Benzodiazepines | Day | 0.01 | As above |
| Hypnotics and sedatives | Day | 0.01 | As above |
| Mood stabilizers | Day | 0.24 | As above |
| **Neuropsychological assessment** | Assessment | 69.12 | l'Assurance Maladie: <http://www.ameli.fr/accueil-de-la-ccam/trouver-un-acte/fiche-abregee.php?code=ALQP006>, Accessed on April 19, 2011. |
| **Institutionalisation cost** | Day | 69.80 | Daily institutionalisation cost based on long-term institutional care (nursing home) costs: taken from the mean of the average cost borne by nursing home residents in Paris (2780 € per month), Banlieue Parisienne (2161 € per month) and Province (1591 € per month) in 2011 values, calculated as 71.58 € per day in Les Maisons de Retraite.fr: <http://www.lesmaisonsderetraite.fr/maisons-de-retraite/la-tarification.htm>, Accessed on September 26, 2011 |

**(b) Germany**

| **Unit cost** | **Unit of measurement** | **Unit cost (€; 2010 values)** | **Source** |
| --- | --- | --- | --- |
| **Caregiver informal care costs** | | | |
| Caregiver time: lost production | Hour | 24.09 | Average gross wage for country. Average full-time employee earns €3,227 per month after tax. Extra payments not included (2010). Average usual weekly hours worked on the main job are 35.7 hours (2009).  Statistische Bundesamt Deutschland: <http://www.destatis.de/jetspeed/portal/cms/Sites/destatis/Internet/EN/Content/Statistics/VerdiensteArbeitskosten/EarningsEconomicActivity/Current__EconomicActivity.psml>, Accessed on September 26, 2011.  OECD: <http://stats.oecd.org/Index.aspx?DataSetCode=AVE_HRS>, Accessed on September 26, 2011.  OECD Employer social security contributions: [www.oecd.org/ctp/taxdatabase](http://www.oecd.org/ctp/taxdatabase), Accessed on September 26, 2011. |
| Caregiver time: lost leisure time | Hour | 8.43 | 35% of average gross wage |
| Cost of caregiver missing work | Hour | 24.09 | As average gross wage |
| **Healthcare resource use costs** | | | |
| *Nights in hospital* |  |  |  |
| Geriatric ward | Night | 236.63 | Fallpauschalenkatalog 2010: <http://www.g-drg.de/cms/Media/Files/G-DRG-System_2011/Fallpauschalen-Katalog_2010_PDF/%28language%29/ger-DE>, Accessed on September 26, 2011. |
| Psychiatric ward | Night | 487.34 | Source as above |
| Internal medicine ward | Night | 320.07 | Source as above |
| Surgical ward | Night | 804.26 | Source as above |
| Other | Night | 462.07 | Assumed as average of above costs. |
| *Emergency room (ER) visits* | Visit | 50.14 | KBV: <http://www.kbv.de/ebm2011/ebmgesamt.htm>, Accessed on September 26, 2011. |
| *Outpatient visits* |  |  |  |
| General practitioner | Visit | 18.13 | Krauth C (2008) Health care economics analysis of early diagnosis. *Laryngorhinootologie* **87**(Suppl 1),S81-93. |
| Geriatrician | Visit | 61.00 | Ehret R, Balzer-Geldsetzer M, Reese JP, Dodel I, Becker E, Christopher A, Friedrich H, Kraemer S, Lüer W, Müngersdorf M, Puzich R, Rohr A, Schultes-Platzek I, Siefjediers V, Tiel-Wilck K, Oertel WH, Dodel R (2009) Direkte Kosten der Parkinson-Behandlung. *Nervenarzt*, **80**,452-8. |
| Neurologist | Visit | 61.00 | Ehret R, Balzer-Geldsetzer M, Reese JP, Dodel I, Becker E, Christopher A, Friedrich H, Kraemer S, Lüer W, Müngersdorf M, Puzich R, Rohr A, Schultes-Platzek I, Siefjediers V, Tiel-Wilck K, Oertel WH, Dodel R (2009) Direkte Kosten der Parkinson-Behandlung. *Nervenarzt*, **80**,452-8. |
| Psychiatrist | Visit | 17.37 | Krauth C (2008) Health care economics analysis of early diagnosis. *Laryngorhinootologie* **87**(Suppl 1),S81-93. |
| Physiotherapist | Visit | 26.48 | VDEK: <http://www.vdek.com/vertragspartner/sonstige-vertragspartner/heilmittelerbringer/zulassung/verguetungslisten-west/Verguetungsvereinbarung_west_2011.pdf>, Accessed on September 26, 2011. |
| Occupational therapist | Visit | 26.48 | VDEK: <http://www.vdek.com/vertragspartner/sonstige-vertragspartner/heilmittelerbringer/zulassung/verguetungslisten-west/Verguetungsvereinbarung_west_2011.pdf>, Accessed on September 26, 2011. |
| Social worker | Visit | 32.58 | Based on cost for UK. Assumed 30 minute visit.  Unit Costs of Health and Social Care 2010: [www.pssru.ac.uk/pdf/uc/uc2010/uc2010_s01.pdf](http://www.pssru.ac.uk/pdf/uc/uc2010/uc2010_s01.pdf), Accessed on September 26, 2011. |
| Psychologist | Visit | 55.42 | 50 minute consultation  KBV: <http://www.kbv.de/ebm2011/ebmgesamt.htm>, Accessed on September 26, 2011. |
| Other health care professional | Visit | 37.99 | Assumed as average of above costs, excluding social worker visits. |
| **Community care services** |  |  |  |
| District nurse | Visit/hour | 12.31 | KBV: <http://www.kbv.de/ebm2011/ebmgesamt.htm>, Accessed on September 26, 2011.  EBM Code 01422 (380 points) Erstverordnung von Behandlungsmaßnahmen zur psychiatrischen häuslichen Krankenpflege (first prescription of treatment with psychiatric nursing activity at home). For Germany, district nurse was interpreted as "Gemeindeschwester/Pflegedienst" |
| Home support worker | Visit/hour | 5.02 | [Kobelt](http://www.ncbi.nlm.nih.gov/sites/entrez?cmd=search&db=PubMed&term=%20Kobelt%20G%5Bauth%5D) G, Berg J, Lindgren P, Elias WG, Flachenecker P, Freidel M, Konig N, Limmroth V, Straube E (2006) Cost and quality of life of multiple sclerosis in Germany. *Eur J Health Econ* **7**(Suppl 2),S34-44. |
| Home-delivered meals | Visit/hour | 10.70 | Mean cost of home delivered food in Berlin/Munich/Hamburg, based on home delivery web site. Minimum order value  BRKG: <http://www.gesetze-im-internet.de/bundesrecht/brkg_2005/gesamt.pdf>, Accessed on September 26, 2011. |
| Transportation | Visit/hour | 4.00 | Assumed 10 km distance to community care center (*2). Cost equals €0.2/km.  http://www.gesetze-im-internet.de/bundesrecht/brkg_2005/gesamt.pdf |
| Day care | Visit/hour | 19.44 | KBV: <http://www.kbv.de/ebm2011/ebmgesamt.htm>, Accessed on September 26, 2011.  EBM Code 01410 (600 points) Besuch |
| Other services | Visit/hour | 10.30 | Assumed as average of above. |
| **Cost of medication for other disorders** |  |  |  |
| Depression | Day | 0.27 | Average cost of three most common medications for the disease  Medication lists: IMS MIDAS quarterly sales, for 2010 using PADDS database viewer, identified based on market share per DOT  Medication costs: Lauer Taxe online, pharmaceutical database for Germany, price from January 15, 2012; <http://taxe.lauer-fischer.de/>; WHO ATC/DDD  DDD: ATC/DDD Index 2011: <http://www.whocc.no/atc_ddd_index>, Accessed on September 26, 2011. |
| Epilepsy | Day | 1.01 | As above |
| Stroke | Day | 0.18 | As above |
| Ischemic cardiac | Day | 0.16 | As above |
| Hypertension | Day | 0.20 | As above |
| Diabetes mellitus | Day | 0.20 | As above |
| Hypercholesterolemia | Day | 0.12 | As above |
| Urinary tract disorder | Day | 0.44 | As above |
| Obstructive pulmonary | Day | 0.80 | As above |
| **AD medications** |  |  |  |
| Donepezil | Day | 5.40 | Medication costs: Rote Liste: <http://www.rote-liste.de/Online/jumpsearch>, Accessed on September 26, 2011.  Medication doses: ATC/DDD Index 2011: <http://www.whocc.no/atc_ddd_index>, Accessed on September 26, 2011. |
| Galantamine | Day | 4.36 | Sources as above |
| Memantine | Day | 3.70 | Sources as above |
| Rivastigmine | Day | 3.96 | Sources as above |
| Other AD medication | Day | 4.35 | Assumed as average of above costs. |
| **Psychiatric/hypnotic medication** |  |  |  |
| Antidepressants | Day | 0.27 | Average cost of three most common medications  Medication lists: IMS MIDAS quarterly sales, for 2010 using PADDS database viewer, identified based on market share per DOT  Medication costs: Lauer Taxe online, pharmaceutical database for Germany, price from January 15, 2012; <http://taxe.lauer-fischer.de/>; WHO ATC DDD  ATC/DDD Index 2011. <http://www.whocc.no/atc_ddd_index>, Accessed on September 26, 2011. |
| Antipsychotics | Day | 1.53 | As above plus DDD adjusted for elderly dosing: BNF 62 and *Curr Med Res Opin*.1992,**12**, p.615. |
| Anxiolytics | Day | 0.10 | As for antidepressants plus DDD adjusted for elderly dosing: BNF 62 and *Therapie* 1989, **44**, pp.219-22. |
| Benzodiazepines | Day | 0.24 | As for antidepressants plus DDD adjusted for elderly dosing: BNF 62 |
| Hypnotics and sedatives | Day | 0.22 | As for antidepressants plus DDD adjusted for elderly dosing: BNF 62 |
| Mood stabilizers | Day | 1.51 | As for antidepressants plus DDD adjusted for elderly dosing: BNF 62 |
| **Neuropsychological assessment** | Assessment | 24.14 | Kassenärztliche Bundesvereinigung: <http://www.kbv.de/ebm2011/ebmgesamt.htm>,  Accessed on April 19, 2011. |
| **Institutionalisation cost** | Day | 108.52 | Daily institutionalisation cost based on long-term institutional care (nursing home) costs: Mean of costs for care levels 1, 2 and 3 (Pflegestufe 1 [€87.86], Pflegestufe 2 [€101.26] and Pflegestufe 3 [€111.20]) for 2005 (€100.11) in Kostenstruktur in Pflegeheimen: http://www.muenchnerpflegeboerse.de/downloads/beschluss061012_kostenstruktur_pflegeheime.pdf?PHPSESSID=af790a7706c96d177705c72a27688bd2). Accessed on September 26, 2011 |

**(c) UK**

| **Unit**  **cost** | **Unit of measurement** | **Unit cost**  **(£; 2010 values)** | **Unit cost (€; 2010 values)** | **Source** |
| --- | --- | --- | --- | --- |
| **Caregiver informal care costs** | | | | |
| Caregiver time: lost production | Hour | 15.65 | 18.25 | Office for national statistics: <http://www.statistics.gov.uk/STATBASE/ssdataset.asp?vlnk=7427>, Accessed on September 26, 2011.  OECD Employer social security contributions: [www.oecd.org/ctp/taxdatabase](http://www.oecd.org/ctp/taxdatabase), Accessed on September 26, 2011. |
| Caregiver time: lost leisure time | Hour | 5.48 | 6.39 | 35% of average gross wage |
| Cost of caregiver missing work | Hour | 15.65 | 18.25 | As average gross wage |
| **Healthcare resource use costs** | | | | |
| *Nights in hospital* |  |  |  |  |
| Geriatric ward | Night | 297.74 | 347.19 | Cost per bed-day, inpatient stay elderly person.  Unit Costs of Health and Social Care 2010: [www.pssru.ac.uk/pdf/uc/uc2010/uc2010_s01.pdf](http://www.pssru.ac.uk/pdf/uc/uc2010/uc2010_s01.pdf), Accessed on September 26, 2011. |
| Psychiatric ward | Night | 297.74 | 347.19 | As above |
| Internal medicine ward | Night | 297.74 | 347.19 | As above |
| Surgical ward | Night | 297.74 | 347.19 | As above |
| Other | Night | 297.74 | 347.19 | Assumed as average of above costs. |
| *Emergency room (ER) visits* | Visit | 63.68 | 74.26 | Unit Costs of Health and Social Care 2010: [www.pssru.ac.uk/pdf/uc/uc2010/uc2010_s01.pdf](http://www.pssru.ac.uk/pdf/uc/uc2010/uc2010_s01.pdf), Accessed on September 26, 2011. |
| *Outpatient visits* |  |  |  |  |
| General practitioner | Visit | 36.58 | 42.66 | Per surgery consultation averaging 11.7 minutes  Unit Costs of Health and Social Care 2010: [www.pssru.ac.uk/pdf/uc/uc2010/uc2010_s01.pdf](http://www.pssru.ac.uk/pdf/uc/uc2010/uc2010_s01.pdf), Accessed on September 26, 2011. |
| Geriatrician | Visit | 36.33 | 42.36 | Average of medical and psychiatric consultant; assume 10 minute visit.  Unit Costs of Health and Social Care 2010: [www.pssru.ac.uk/pdf/uc/uc2010/uc2010_s01.pdf](http://www.pssru.ac.uk/pdf/uc/uc2010/uc2010_s01.pdf), Accessed on September 26, 2011. |
| Neurologist | Visit | 36.33 | 42.36 | Average of medical and psychiatric consultant; assume 10 minute visit.  Unit Costs of Health and Social Care 2010: [www.pssru.ac.uk/pdf/uc/uc2010/uc2010_s01.pdf](http://www.pssru.ac.uk/pdf/uc/uc2010/uc2010_s01.pdf), Accessed on September 26, 2011. |
| Psychiatrist | Visit | 47.93 | 55.89 | £283 per psychiatrist hour patient contact adjusted for 10 minute visit.  Unit Costs of Health and Social Care 2010: [www.pssru.ac.uk/pdf/uc/uc2010/uc2010_s01.pdf](http://www.pssru.ac.uk/pdf/uc/uc2010/uc2010_s01.pdf), Accessed on September 26, 2011. |
| Physiotherapist | Visit | 19.05 | 22.21 | Mean of community care based and hospital based; assumed 30 minute visit.  Unit Costs of Health and Social Care 2010: [www.pssru.ac.uk/pdf/uc/uc2010/uc2010_s01.pdf](http://www.pssru.ac.uk/pdf/uc/uc2010/uc2010_s01.pdf), Accessed on September 26, 2011. |
| Occupational therapist | Visit | 21.34 | 24.88 | Mean of community care based and hospital based; assumed 30 minute visit.  Unit Costs of Health and Social Care 2010: [www.pssru.ac.uk/pdf/uc/uc2010/uc2010_s01.pdf](http://www.pssru.ac.uk/pdf/uc/uc2010/uc2010_s01.pdf), Accessed on September 26, 2011. |
| Social worker | Visit | 27.94 | 32.58 | Assumed 30 minute visit  Unit Costs of Health and Social Care 2010: [www.pssru.ac.uk/pdf/uc/uc2010/uc2010_s01.pdf](http://www.pssru.ac.uk/pdf/uc/uc2010/uc2010_s01.pdf), Accessed on September 26, 2011. |
| Psychologist | Visit | 41.15 | 47.99 | Assumed 30 minute visit  Unit Costs of Health and Social Care 2010: [www.pssru.ac.uk/pdf/uc/uc2010/uc2010_s01.pdf](http://www.pssru.ac.uk/pdf/uc/uc2010/uc2010_s01.pdf), Accessed on September 26, 2011. |
| Other health care professional | Visit | 33.33 | 38.87 | Assumed average of above costs. |
| **Community care services** |  |  |  |  |
| District nurse | Visit/hour | 24.39 | 28.44 | Per home visit  Unit Costs of Health and Social Care 2010: [www.pssru.ac.uk/pdf/uc/uc2010/uc2010_s01.pdf](http://www.pssru.ac.uk/pdf/uc/uc2010/uc2010_s01.pdf), Accessed on September 26, 2011. |
| Home support worker | Visit/hour | 21.75 | 25.36 | £21.4 one hour per week of local authority-organized home care.  Unit Costs of Health and Social Care 2010: [www.pssru.ac.uk/pdf/uc/uc2010/uc2010_s01.pdf](http://www.pssru.ac.uk/pdf/uc/uc2010/uc2010_s01.pdf), Accessed on September 26, 2011. |
| Home-delivered meals | Visit/hour | 5.11 | 5.96 | The average cost of providing meals to adults and older people is £4.90 per meal or £24.00 per week, equating to just under five meals per person  Personal Social Services Expenditure and Unit Costs England: 2008-9-2009.  <http://www.ic.nhs.uk/webfiles/publications/009_Social_Care/pss0910expfinal/pss0910expfinal_update_070311/Personal_Social_Services_Expenditure_Report%202009_10.pdf>, Accessed on September 26, 2011. |
| Transportation | Visit/hour | 22.44 | 26.17 | Hospital Travel Cost Scheme - Outpatient, Hospital Travel Cost Scheme -other; Patient Transport Services - Outpatient; Patient Transport Services - other  National Schedule of Reference Costs 2008-09 for NHS Trusts: <http://www.dh.gov.uk/en/Publicationsandstatistics/Publications/PublicationsPolicyAndGuidance/DH_123459>, Accessed on September 26, 2011. |
| Day care | Visit/hour | 11.18 | 13.04 | £33 per user session (a session is defined as either a morning, afternoon, or evening at the day care facility). Assume three hours for one session. So day care per hour  Unit Costs of Health and Social Care 2010: [www.pssru.ac.uk/pdf/uc/uc2010/uc2010_s01.pdf](http://www.pssru.ac.uk/pdf/uc/uc2010/uc2010_s01.pdf), Accessed on September 26, 2011. |
| Other services | Visit/hour | 16.97 | 19.79 | Assumed as average of above costs. |
| **Cost of medication for other disorders** |  |  |  |  |
| Depression | Day | 0.081 | 0.09 | Average cost of three most common treatments  Medication lists: IMS MIDAS quarterly sales using PADDS database viewer, market share data per DOT for UK was used for year 2010  Medication costs: BNF 62 (September, 2011) (<http://www.medicinescomplete.com/mc/bnf/current/index.htm>, Accessed on September 26, 2011) and ATC/DDD Index 2011 (<http://www.whocc.no/atc_ddd_index>, Accessed on September 26, 2011) |
| Epilepsy | Day | 0.315 | 0.37 | As above |
| Stroke | Day | 0.060 | 0.07 | As above |
| Ischemic cardiac | Day | 0.019 | 0.02 | As above |
| Hypertension | Day | 0.099 | 0.12 | As above |
| Diabetes mellitus | Day | 0.268 | 0.31 | As above |
| Hypercholesterolemia | Day | 0.604 | 0.70 | As above |
| Urinary tract disorder | Day | 0.319 | 0.37 | As above |
| Obstructive pulmonary | Day | 0.328 | 0.38 | Average based on Table 1 in Britton M (2003) The burden of COPD in the UK: results from the Confronting COPD survey. *Respir Med* **97**(Suppl C),S71-9; values inflated to 2010 values. |
| **AD medications** |  |  |  |  |
| Donepezil | Day | 3.05 | 3.56 | Medication costs: BNF 61 (<http://www.medicinescomplete.com/mc/bnf/current/index.htm>, Accessed on September 26, 2011)  Medication doses: ATC/DDD Index 2011(<http://www.whocc.no/atc_ddd_index>, Accessed on September 26, 2011. |
| Galantamine | Day | 2.32 | 2.71 | As above |
| Memantine | Day | 2.34 | 2.73 | As above |
| Rivastigmine | Day | 2.26 | 2.64 | As above |
| Other AD medication | Day | 2.49 | 2.90 | Assumed as average of above costs. |
| **Psychiatric/hypnotic medication** |  |  |  |  |
| Antidepressants | Day | 0.087 | 0.10 | Average cost of three most common treatments  Medication lists: IMS MIDAS quarterly sales using PADDS database viewer, market share data per DOT for UK was used for year 2010  Medication costs: BNF 62 (September, 2011) (http://www.medicinescomplete.com/mc/bnf/current/index.htm) & ATC/DDD Index 2011(<http://www.whocc.no/atc_ddd_index/>, Accessed on September 26, 2011) |
| Antipsychotics | Day | 1.438 | 1.68 | As above and DDD adjusted for elderly dosing BNF 62 |
| Anxiolytics | Day | 0.117 | 0.14 | As above and DDD adjusted for elderly dosing BNF 62 |
| Benzodiazepines | Day | 0.044 | 0.05 | As above and DDD adjusted for elderly dosing BNF 62 |
| Hypnotics and sedatives | Day | 0.049 | 0.06 | As above and DDD adjusted for elderly dosing BNF 62 |
| Mood stabilizers | Day | 0.670 | 0.78 | As above and DDD adjusted for elderly dosing BNF 62 |
| **Neuropsychological assessment** | Assessment | 83.76 | 97.67 | National Schedule of Reference Costs Year : '2008–09' - NHS Trusts Direct Access: Diagnostic Services – other test |
| **Institutionalisation cost** | Day | 99.15 | 115.62 | Daily institutionalization cost based on long-term institutional care (private nursing home) costs: £683 per week (2009-2010 values), calculated as £97.57 per day, in Unit Costs of Health and Social Care 2010: [www.pssru.ac.uk/pdf/uc/uc2010/uc2010_s01.pdf](http://www.pssru.ac.uk/pdf/uc/uc2010/uc2010_s01.pdf), Accessed on September 26, 2011 |
|  |  |  |  |  |

All information except institutionalisation costs is reprinted from Supplementary Table 1 in Journal of Alzheimer’s Disease, Volume 36, Wimo A, Reed CC, Dodel R, Belger M, Jones RW, Happich M, Argimon JM, Bruno G, Novick D, Vellas B, Haro JM, The GERAS Study: A Prospective Observational Study of Cost and Resource Use in Community Dwellers with Alzheimer’s Disease in Three European Countries – Study Design and Baseline Findings, 385-399, Copyright (2013), with permission from IOS Press. The publication is available at IOS Press through <http://dx.doi.org/10.3233/JAD-122392>

All UK costs in pounds sterling were converted to euros using the conversion rate: £1 = €1.1661 (calculated as the monthly exchange rate average for 2010, <http://www.x-rates.com/d/EUR/GBP/hist2010.html>, Accessed on June 22, 2012).

(AMK: Actes pratiqués par le masseur-kinésithérapeute; ATC: Anatomical, therapeutic and chemical; ATIH: Agence Technique de l'information sur l’Hospitalisation; BNF: British National Formulary; BRKG: Bundesreisekostengesetz; CIDJ: Centre d’Information et de Documentation Jeunesse; DOT: duration of treatment; DDD: defined daily dose; EBM: Einheitlicher Bewertungsmaßstab; EHPAD: Etablissements d'Hébergement pour Personnes Agées Dépendantes; INSEE: National Institute of Statistics and Economic Studies - France; KBV: Kassenärztliche Bundesvereinigung; NGAP: Nomenclature générale des actes professionnels; NHS: National Health Service; OECD: The Organisation for Economic Co-operation and Development; Smic: salaire minimum interprofessionnel de croissance; SSIAD: Services de Soins Infirmiers à Domicile; VDEK: Verband der Ersatzkassen e. V.; WHO: World Health Organisation)
