# Supplementary material for: Determinants of time to institutionalisation and related healthcare and societal costs in a community-based cohort of patients with Alzheimer’s disease dementia
Source: Eur J Health Econ. 2018 Sep 3;20(3):343–55. doi: 10.1007/s10198-018-1001-3 (PMC6438944; doi:10.1007/s10198-018-1001-3)
Supplement: Supplementary file 4 — Supplementary material 4 (DOCX 29 KB) [file 10198_2018_1001_MOESM4_ESM.docx]

**Title:** Determinants of time to institutionalisation and related healthcare and societal costs in a community-based cohort of patients with Alzheimer’s disease dementia

**Authors:** Mark Belger, Josep Maria Haro, Catherine Reed, Michael Happich, Josep Maria Argimon, Giuseppe Bruno, Richard Dodel, Roy W. Jones, Bruno Vellas, Anders Wimo

**Corresponding author:** Mark Belger, Erl Wood Manor, Sunninghill Road, Windlesham, Surrey, GU20 6PH, email: [belger_mark@lilly.com](mailto:belger_mark@lilly.com)

**Online Resource 4** Cost estimates (in GBP) from the log-normal regression models of the association between costs and time to institutionalisation in the UK

|  | **Estimate** | **Standard error** | ***p* value** |
| --- | --- | --- | --- |
| ***Total societal costs^a^*** |  |  |  |
| Intercept | 2947.12 | 105.08 | <0.0001 |
| Time to institutionalisation | -460.12 | 65.98 | <0.0001 |
| Time to institutionalisation^2^ | 43.99 | 10.99 | <0.0001 |
| Time to institutionalisation^3^ | -1.57 | 0.51 | 0.002 |
| ***Total patient costs^a^*** |  |  |  |
| Intercept | 1807.16 | 65.89 | <0.0001 |
| Time to institutionalisation | -464.25 | 41.37 | <0.0001 |
| Time to institutionalisation^2^ | 48.39 | 6.89 | <0.0001 |
| Time to institutionalisation^3^ | -1.64 | 0.32 | <0.0001 |
| ***Patient healthcare costs^a^*** |  |  |  |
| Intercept | 198.91 | 12.14 | <0.0001 |
| Time to institutionalisation | -10.23 | 4.67 | 0.03 |
| Time to institutionalisation^2^ | 0.38 | 0.34 | 0.27 |

Time to institutionalisation (Pre-Inst in equations below) is in years

^a^The superscripts 2 and 3 refer to the quadratic and cubic terms, respectively, of the variable ‘time to institutionalisation’

Estimates can be converted into the following equations:

EQ1: Total societal costs (£) = 2947.12 – (460.12 Pre-Inst) + (43.99 Pre-Inst^2^) – (1.57 Pre-Inst^3^)

EQ2: Total patient costs (£) = 1807.16 – (464.25 Pre-Inst) + (48.39 Pre-Inst^2^) – (1.64 Pre-Inst^3^)

EQ3: Patient healthcare costs (£) = 198.91 – (10.23 Pre-Inst) + (0.38 Pre-Inst^2^)
